# Supplementary material for: Non-O ABO blood group genotypes differ in their associations with Plasmodium falciparum rosetting and severe malaria
Source: PLoS Genet. 2023 Sep 14;19(9):e1010910. doi: 10.1371/journal.pgen.1010910 (PMC10522014; doi:10.1371/journal.pgen.1010910)
Supplement: S1 Table — (PDF) [file pgen.1010910.s001.pdf]

S1 Table: The general characteristics of children recruited to the case-control study by *ABO* genotype

ABO genotype		*OO (%)*	*AO (%)*	*AA (%)*	*BO (%)*	*BB (%)*	*AB (%)*	*p* value
**Disease category**	Controls n=3872	2126 (54.9)	810 (20.9)	83 (2.1)	683 (17.6)	55 (1.4)	115 (3.0)	Reference
	All SM n=1398	623 (44.6)	306 (21.9)	37 (2.6)	337 (24.1)	34 (2.4)	61 (4.4)	<0.001
	CM n=716	326 (45.5)	166 (23.2)	22 (3.1)	160 (22.3)	16 (2.2)	26 (3.6)	0.001
	SMA n=415	181 (43.6)	97 (23.4)	9 (2.2)	99 (23.9)	10 (2.4)	19 (4.6)	<0.001
	RD n=417	181 (43.4)	101 (24.2)	13 (3.1)	99 (23.7)	11 (2.6)	12 (2.9)	<0.001
	Died n=116	52 (44.8)	33 (28.4)	2 (1.7)	23 (19.8)	2 (1.7)	4 (3.4)	0.339
**Gender (controls)**	Males	1075 (55.0)	397 (20.3)	31 (1.6)	353 (18.1)	26 (1.3)	71 (3.6)	
	Females	1051 (54.8)	413 (21.5)	52 (2.7)	330 (17.2)	29 (1.5)	44 (2.3)	0.025
**(cases)**	Males	332 (46.4)	155 (21.3)	16 (2.2)	161 (22.5)	19 (2.7)	35 (4.9)	
	Females	291 (42.6)	154 (22.6)	21 (3.1)	176 (25.8)	15 (2.2)	26 (3.8)	0.401
**Ethnic group (controls)**	Giriama	964 (53.7)	377 (21.0)	38 (2.1)	336 (18.7)	24 (1.3)	55 (3.1)	
	Chonyi	796 (57.6)	291 (21.1)	34 (2.5)	209 (15.1)	16 (1.2)	36 (2.6)	
	Kauma	232 (52.1)	91 (20.5)	6 (1.4)	95 (21.4)	8 (1.8)	13 (2.9)	
	Others	134 (53.4)	51 (20.3)	5 (2.0)	43 (17.1)	7 (2.8)	11(4.4)	0.117
**(cases)**	Giriama	372 (44.2)	178 (21.2)	24 (2.9)	209 (24.9)	23 (2.7)	35 (4.2)	
	Chonyi	144 (45.7)	73 (23.2)	3 (1.0)	80 (25.4)	7 (2.2)	8 (2.5)	
	Kauma	36 (38.7)	22 (23.7)	4 (4.3)	19 (20.4)	2 (2.2)	10 (10.8)	
	Others	71 (47.7)	33 (22.2)	6 (4.0)	29 (19.5)	2 (1.3)	8 (5.4)	0.102
**Sickle genotype (controls)**	AA	1779 (54.7)	693 (21.3)	67 (2.1)	578 (17.8)	43 (1.3)	92 (2.8)	
	AS	328 (55.9)	109 (18.6)	16 (2.7)	99 (16.9)	12 (2.0)	23 (3.9)	
	SS	19 (57.6)	8 (24.2)	0 (0.0)	6 (18.2)	0 (0.0)	0 (0.0)	0.496
**(cases)**	AA	615 (44.7)	303 (22.0)	37 (2.7)	329 (23.9)	33 (2.4)	58 (4.2)	
	AS	6 (30.0)	2 (10.0)	0 (0.0)	8 (40.0)	1 (5.0)	3 (15.0)	0.058
	SS	-	-	-	-	-	-	
**α+thalassaemia (controls)**	αα/αα	725 (54.7)	271 (20.5)	36 (2.7)	226 (17.1)	23 (1.7)	44 (3.3)	
	-α/αα	1058 (55.2)	400 (20.9)	34 (1.8)	345 (18)	22 (1.2)	58 (3.0)	
	-α/-α	342 (54.6)	137 (21.9)	13 (2.1)	112 (17.9)	10 (1.6)	13 (2.1)	0.584
**(cases)**	αα/αα	247 (46.3)	112 (21.0)	14 (2.6)	131 (24.5)	10 (1.9)	20 (3.8)	
	-α/αα	272 (42.8)	146 (23.0)	18 (2.8)	153 (24.1)	13 (2.0)	34 (5.4)	
	-α/-α	76 (46.9)	33 (20.4)	3 (1.9)	37 (22.8)	7 (4.3)	6 (3.7)	0.650
**Age in months Median (IQR)***	Controls	6 (5-8)	7 (5-9)	7 (5-9)	6 (5-8)	6 (4-8)	6 (5-9)	0.285
	Cases	28 (17-43)	29 (16-43)	33 (21-49)	30 (17-46)	31 (15-47)	31 (19-44)	0.817

The Pearson's Chi square test (or Fisher's exact test when numbers in any category <10) was used to test for differences in the distribution of *ABO* genotypes across categorical variables of gender, ethnic group and HbS and α^{+}thalassaemia genotype while the Kruskal-Wallis test was used to test for differences in age (as a continuous variable) by *ABO* genotype in controls and cases separately. IQR, interquartile range. For severe malaria, including specific clinical sub-types (CM, cerebral malaria; SMA, severe malarial anaemia; RD, respiratory distress & mortality), comparisons were made to community controls used as the reference group. 77 controls and 5 cases had missing *ABO* genotypes.
