# Supplementary material for: Non-O ABO blood group genotypes differ in their associations with Plasmodium falciparum rosetting and severe malaria
Source: PLoS Genet. 2023 Sep 14;19(9):e1010910. doi: 10.1371/journal.pgen.1010910 (PMC10522014; doi:10.1371/journal.pgen.1010910)
Supplement: S2 Table — (PDF) [file pgen.1010910.s002.pdf]

S2 Table: *P. falciparum* parasite density by *ABO* genotype in severe malaria cases

ABO genotype	*Number of patients*[†]	*Parasite density/*µL (95% CI)	*p* value
OO	612	62550 (50834 – 74265)	Reference
AO	301	57406 (42096 – 72717)	0.605
AA	37	58246 (14006 – 102486)	0.858
BO	335	67952 (50723 – 85181)	0.605
BB	33	72630 (14288 – 130972)	0.723
AB	61	52147 (21218 – 83076)	0.567
Non-O	767	61956 (51601 – 72310)	0.994

Differences in parasite density by *ABO* genotype were tested by linear regression with adjustment for confounding by HbAS, ethnicity and gender. Comparisons were made between *ABO* genotype *OO* and individual non-*O* genotypes (*AO, AA, BO, BB & AB*) or all non-*O* genotypes combined.

[†]Data were available for 1379 of the 1398 cases successfully genotyped for *ABO*. 17 cases had missing parasite density data and 2 had missing HbAS data.
