# Supplementary material for: Non-O ABO blood group genotypes differ in their associations with Plasmodium falciparum rosetting and severe malaria
Source: PLoS Genet. 2023 Sep 14;19(9):e1010910. doi: 10.1371/journal.pgen.1010910 (PMC10522014; doi:10.1371/journal.pgen.1010910)
Supplement: S3 Table — (PDF) [file pgen.1010910.s003.pdf]

S3 Table: Comparing Odds ratio differences for specific severe malaria syndromes between single dose and double dose non-*O* genotypes using the Wald test

Case Phenotype	ABO genotype	No Cases/Controls	Odds Ratio comparisons	Wald test *p* value
All CM	*AO vs AA*	166/810; 22/83	1.29/1.60	0.422
	BO vs BB	160/683; 16/55	1.45/1.90	0.396
	AO vs AB	166/810; 26/115	1.29/1.47	0.595
	BO vs AB	160/683; 26/115	1.45/1.47	0.951
All SMA	*AO vs AA*	97/810; 9/83	1.35/1.18	0.736
	BO vs BB	99/683; 10/55	1.62/1.71	0.896
	AO vs AB	97/810; 19/115	1.35/2.05	0.137
	BO vs AB	99/683; 19/115	1.62/2.05	0.394
All RD	*AO vs AA*	101/810; 13/83	1.41/1.68	0.598
	BO vs BB	99/683; 20/55	1.63/2.01	0.586
	AO vs AB	160/810; 12/115	1.41/1.25	0.715
	BO vs AB	99/683; 12/115	1.63/1.25	0.414
Mortality	*AO vs AA*	33/810; 2/83	1.74/1.00	0.456
	BO vs BB	23/683; 2/55	1.35/1.66	0.785
	AO vs AB	33/810; 4/115	1.74/1.50	0.785
	BO vs AB	23/683; 4/115	1.35/1.50	0.849

The Wald test was used to compare odds ratio differences between single dose and double dose non-*O* genotypes to test the hypothesis that double dose non-*O* genotypes (*AA, AB, BB*) are associated with a higher risk of severe malaria than single dose non-*O* genotypes (*AO*, *BO*). Odds ratios for severe malaria and specific severe malaria syndromes cerebral malaria (CM), severe malarial anaemia (SMA) and respiratory distress (RD), and malaria-specific mortality were determined following a fixed-effects logistic regression model comparing genotype frequencies between the non-*O* genotypes (*AO*, *AA*, *AB*, *BO*, *BB*) to the reference *OO* genotype with adjustments for self-reported ethnicity, gender, α^+thalassaemia and HbAS. SM: Severe malaria; OR: Odds Ratio; LCI: Lower Confidence Interval (95%); UCI: Upper Confidence Interval. †Adjusted for HbS, α+thalassaemia, gender, ethnicity and interaction (HbS and α+thalassaemia).
