# Supplementary material for: Non-O ABO blood group genotypes differ in their associations with Plasmodium falciparum rosetting and severe malaria
Source: PLoS Genet. 2023 Sep 14;19(9):e1010910. doi: 10.1371/journal.pgen.1010910 (PMC10522014; doi:10.1371/journal.pgen.1010910)
Supplement: S4 Table — (PDF) [file pgen.1010910.s004.pdf]

S4 Table: A comparison of the odds ratio differences for severe malaria between single dose and double dose non-*O* genotypes using logistic regression with single dose non-*O* genotypes *AO/BO* as reference

			Crude				Adjusted†			
**Case Phenotype**	**No. Cases/controls**	***ABO* genotype**	**OR**	**LCI**	**UCI**	***p* value**	**OR**	**LCI**	**UCI**	***p* value**
All SM	306/810	*AO*	1				1			Reference
	37/83	*AA*	1.18	0.78	1.78	0.428	1.15	0.74	1.78	0.528
	61/115	*AB*	1.40	1.00	1.97	0.049	1.53	1.07	2.18	0.020
All SM	337/683	*BO*	1				1			Reference
	34/55	*BB*	1.25	0.80	1.96	0.323	1.26	0.77	2.07	0.351
	61/115	*AB*	1.08	0.77	1.51	0.674	1.17	0.82	1.68	0.384

Double dose non-*O* genotype odds ratios for severe malaria were generated following a fixed-effects logistic regression model comparing genotype frequencies between the non-*O* double dose genotypes (*AA/AB* or *BB/AB*) to the reference non-*O* single dose genotypes (*AO* or *BO*) with adjustments for self-reported ethnicity, gender, α^+thalassaemia and HbAS. SM: Severe malaria; OR: Odds Ratio; LCI: Lower Confidence Interval (95%); UCI: Upper Confidence Interval. †Adjusted for HbS, α+thalassaemia, gender, ethnicity and interaction (HbS and α+thalassaemia).
