# Supplementary material for: Non-O ABO blood group genotypes differ in their associations with Plasmodium falciparum rosetting and severe malaria
Source: PLoS Genet. 2023 Sep 14;19(9):e1010910. doi: 10.1371/journal.pgen.1010910 (PMC10522014; doi:10.1371/journal.pgen.1010910)
Supplement: S5 Table — (PDF) [file pgen.1010910.s005.pdf]

S5 Table: Cytoadhesion of *P. falciparum* line ItG by *ABO* genotype

**ItG static adhesion to CD36**				
**N**	**Genotype**	**Mean pRBCs bound/mm²**	**95% CI**	***p* value**
51	*OO*	1385.64	1179.40 – 1591.89	-
32	*AO*	1345.61	1087.33 – 1603.89	0.814
6	*AA*	817.42	312.59 – 1322.25	0.073
17	*BO*	1146.66	813.49 – 1479.84	0.246
3	*BB*	1030.24	308.69 – 1751.79	0.386
3	*AB*	779.62	157.27 – 1401.96	0.123
61	*Non-O*	1191.53	1014.74 – 1368.32	0.163
**ItG static adhesion to ICAM-1**				
**N**	**Genotype**	**Mean pRBCs bound/mm²**	**95% CI**	***p* value**
51	*OO*	1640.72	1389.18– 1892.27	-
32	*AO*	1602.70	1286.15 – 1919.25	0. 854
6	*AA*	1567.11	772.38 – 2361.83	0.864
17	*BO*	1356.65	956.95 – 1756.34	0.254
3	*BB*	1296.59	398.11 – 2195.08	0.493
3	*AB*	1030.40	237.05 – 1823.75	0.208
61	*Non-O*	1479.30	1261.03 – 1697.57	0.340

Differences in ItG *P. falciparum* line binding to CD36 and ICAM-1 by *ABO* genotype were tested using multivariate linear regression analysis with adjustment for confounding by HbAS and α^+thalassemia genotypes (including an interaction between HbAS and α^+thalassemia). 112 RBC donor samples were tested once in duplicate over seven experimental days (day 1 n = 13, day 2 n = 8, day 3 n = 12, day 4 n = 1, day 5 n = 55, day 6 n = 15 and day 7 n = 8), therefore, experimental day was included as a co-variate to account for day-to-day variation.
