# Supplementary material for: Non-O ABO blood group genotypes differ in their associations with Plasmodium falciparum rosetting and severe malaria
Source: PLoS Genet. 2023 Sep 14;19(9):e1010910. doi: 10.1371/journal.pgen.1010910 (PMC10522014; doi:10.1371/journal.pgen.1010910)
Supplement: S6 Table — (PDF) [file pgen.1010910.s006.pdf]

S6 Table: ITvar9 PfEMP1 expression by *ABO* genotype

IT/R29 (ITvar9) PfEMP1 expression				
**N**	**Genotype**	**Mean MFI**	**95% CI**	***p* value**
23	*OO*	3412.88	3180.61 – 3645.17	-
18	*AO*	3548.06	3283.57 – 3812.56	0.452
2	*AA*	3714.24	2869.41– 4559.07	0.487
9	*BO*	3325.30	2952.46– 3698.14	0.696
1	*BB*	3753.62	2617.84 – 4889.41	0.562
7	*AB*	3750.16	3317.22 – 4183.10	0.173
37	*Non-O*	3550.45	3372.44 – 3728.45	0.333
**Percentage ITvar9 PfEMP1 expression**				
**N**	**Genotype**	**% pRBCs ITvar9 positive**	**95% CI**	***p* value**
23	*OO*	56.71	53.74 – 59.69	-
18	*AO*	54.92	51.53 – 58.31	0.437
2	*AA*	57.94	47.13 – 68.76	0.824
9	*BO*	53.40	48.62 – 58.17	0.252
1	*BB*	56.40	41.86 – 70.95	0.967
7	*AB*	55.73	50.19– 61.28	0.756
37	*Non-O*	54.95	52.71 – 57.18	0.361

Differences in ITvar9 PfEMP1 expression by *ABO* genotype in the *P. falciparum* IT/R29 rosetting parasite line were tested by multivariate regression analysis with adjustment for confounding by HbAS and α^+thalassemia (including interaction between HbAS and α^+thalassemia). 60 RBC donor samples were tested once in duplicate over two successive experimental days (day 1 n = 30 and day 2 n = 30), therefore, experimental day was included as a co-variate to account for day-to-day variation.
