# Supplementary material for: Non-O ABO blood group genotypes differ in their associations with Plasmodium falciparum rosetting and severe malaria
Source: PLoS Genet. 2023 Sep 14;19(9):e1010910. doi: 10.1371/journal.pgen.1010910 (PMC10522014; doi:10.1371/journal.pgen.1010910)
Supplement: S7 Table — (PDF) [file pgen.1010910.s007.pdf]

S7 Table: General characteristics of the Kilifi longitudinal cohort study by *ABO* genotype

Characteristics		*OO (%)*	*AO (%)*	*AA (%)*	*BO (%)*	*BB (%)*	*AB (%)*	*p* value
**Sample size**	n = 242	135 (55.8)	52 (21.5)	2 (0.8)	44 (18.2)	4 (1.6)	5 (2.1)	
**Gender**	Males	58 (56.3)	21 (20.4)	0 (0.0)	20 (19.4)	2 (1.94)	2 (1.94)	
	Females	77 (55.4)	31 (22.3)	2 (1.4)	24 (17.3)	2 (1.4)	3 (2.2)	0.933
**Ethnic group**	Giriama	118 (56.5)	44 (21.1)	1 (0.5)	39 (18.6)	4 (1.9)	3 (1.4)	
	Chonyi	8 (38.1)	6 (28.5)	1 (4.8)	5 (23.8)	0 (0.0)	1 (4.8)	
	Others	9 (75.0)	2 (16.7)	0 (0.0)	0 (0.0)	0 (0.0)	1 (8.3)	0.120
**Sickle**	AA	124 (58.5)	43 (20.3)	2 (0.9)	38 (17.9)	2 (0.9)	3 (1.4)	
	AS	11 (36.6)	9 (30.0)	0 (0.0)	6 (20.0)	2 (6.67)	2 (6.67)	0.029
**α+thalassaemia**	αα/αα	41 (55.4)	20 (27.0)	1 (1.4)	11 (14.8)	1 (1.4)	0 (0.0)	
	-α/αα	75 (61.5)	16 (13.1)	1 (0.8)	25 (20.5)	2 (1.6)	3 (2.5)	
	-α/-α	19 (41.3)	16 (34.7)	0 (0.0)	8 (17.4)	1 (2.2)	2 (4.4)	0.043
**Age in months**								
**Median (IQR)**		32.2 (16-49)	30 (12-50)	39 (30-49)	39 (20-53)	40 (18-54)	39 (18-49)	<0.001

The Fisher's exact test was used to test for differences in the distribution of *ABO* genotypes across categorical variables of gender, ethnic group, HbS and α^+thalassemia genotypes while the Kruskal-Wallis test was used to test for differences in age (as a continuous variable) by *ABO* genotype. IQR, interquartile range. The significant differences in *ABO* genotypes in relation to HbS genotype, α+thalassaemia genotype and age may be due to the very small numbers in the "double dose" non-*O* genotype categories. Analysis of this dataset therefore included adjustment for these variables.
