# Supplementary material for: Non-O ABO blood group genotypes differ in their associations with Plasmodium falciparum rosetting and severe malaria
Source: PLoS Genet. 2023 Sep 14;19(9):e1010910. doi: 10.1371/journal.pgen.1010910 (PMC10522014; doi:10.1371/journal.pgen.1010910)
Supplement: S8 Table — (PDF) [file pgen.1010910.s008.pdf]

S8 Table: Incidence rate ratios (IRR) for uncomplicated malaria in Kenya by *ABO* genotype

N	Episodes	*ABO* genotype	Crude				Adjusted†			
			IRR	LCI	UCI	*p* value	IRR	LCI	UCI	*p* value
135	528	*OO*	1				1			
52	237	*AO*	1.13	0.80	1.60	0.495	1.26	0.90	1.78	0.181
2	10	*AA*	1.19	0.27	5.31	0.817	1.03	0.26	4.05	0.963
5	19	*AB*	0.81	0.30	2.16	0.672	1.01	0.40	2.56	0.986
44	158	*BO*	0.81	0.56	1.18	0.279	0.95	0.66	1.37	0.787
4	6	*BB*	0.37	0.10	1.38	0.139	0.58	0.16	2.11	0.411
107	430	Non-*O**	0.96	0.72	1.27	0.753	1.09	0.83	1.45	0.532

Incidence rate ratios and 95% confidence intervals were generated using a random effects Poisson regression analysis, either without or †with adjustment for age, season, ethnic group, HbAS and α^+thalassaemia. The analysis also took into account within person clustering of events. Data represent 250, 88.1, 4.5, 10.7, 82.6, & 6.1 child years of follow up for *OO, AO, AA, AB, BO* and *BB* genotypes respectively. * Analysis comparing non-*O* to *OO* was done using a recessive model of inheritance. Abbreviations: IRR, incident rate ratio; LCI: lower 95% confidence interval; UCI: upper 95% confidence interval.
