# Supplementary material for: Non-O ABO blood group genotypes differ in their associations with Plasmodium falciparum rosetting and severe malaria
Source: PLoS Genet. 2023 Sep 14;19(9):e1010910. doi: 10.1371/journal.pgen.1010910 (PMC10522014; doi:10.1371/journal.pgen.1010910)
Supplement: S9 Table — (PDF) [file pgen.1010910.s009.pdf]

S9 Table: Odds ratios (OR) for asymptomatic malaria in Kenya by *ABO* genotype

			Crude					Adjusted[†]			
**N**	**Prevalence[§]**	***ABO* genotype**	**OR**	**LCI**	**UCI**	***p* value**		**OR**	**LCI**	**UCI**	***p* value**
124	44/360 (12%)	*OO*	1					1			
48	24/130 (18.5%)	*AO*	1.63	0.90	2.97	0.110		1.84	1.00	3.38	0.051
2	0/7 (0.0%)	*AA*	-	-	-	-	-	-	-	-	-
5	3/16 (18.8%)	*AB*	1.66	0.20	13.52	0.635		1.77	0.20	15.86	0.749
43	18/127 (14.2%)	*BO*	1.19	0.62	2.27	0.598		1.12	0.56	2.26	0.743
2	2/7 (28.6%)	*BB*	2.88	0.52	15.94	0.225		3.34	0.66	16.91	0.146
100	47/287 (16.4%)	Non-*O**	1.41	0.84	2.36	0.190		1.45	0.85	2.46	0.170

[§]Prevalence: Number of *P. falciparum* positive slides/total slides (%). These data were derived from four cross-sectional surveys as part of the Kilifi longitudinal cohort study carried out in March, July and October 2000 and June 2001. Odds ratios and 95% confidence intervals were generated using a logistic regression analysis, without or [†]with adjustment for age, season, ethnic group and HbAS genotype. The analysis also took into account within person clustering of events. *Analysis comparing non-*O* to blood group *O* was done using a recessive model of inheritance. Abbreviations: OR, odds ratio; LCI: lower 95% confidence interval; UCI: upper 95% confidence interval.
