# Supplementary figures and images for: Non-O ABO blood group genotypes differ in their associations with Plasmodium falciparum rosetting and severe malaria
Source: PLoS Genet. 2023 Sep 14;19(9):e1010910. doi: 10.1371/journal.pgen.1010910 (PMC10522014; doi:10.1371/journal.pgen.1010910)

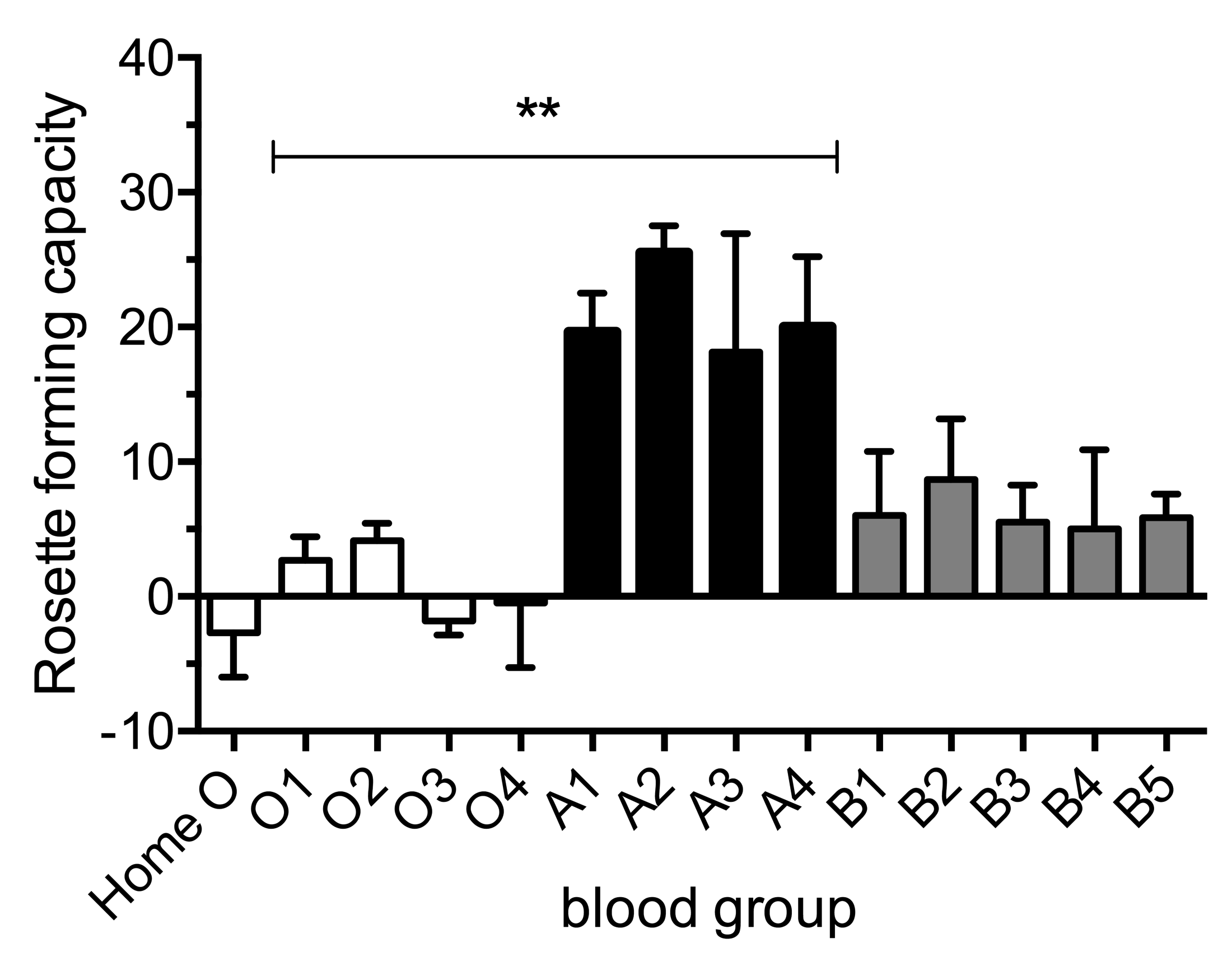

Supplement: S1 Fig — ABO blood group preference assay of the IT/R29 parasite clone was assessed using fluorescently-labelled RBC from 13 donors (4 group O, 4 group A and 5 group B). “Home O” indicates the blood group O donor that was used to culture the parasites. The y-axis shows the difference between the percentage of labelled cells found in rosettes, compared to the percentage found in the mix. Experiments were carried out in triplicate, and the mean and SEM are shown for each donor. For statistical analysis, the triplicate values for each donor were averaged and treated as a single data point, such that n = 4 for groups O and A, and n = 5 for group B. The blood groups were compared using a Kruskal Wallis test with Dunn’s multiple comparisons (** p<0.01). (TIFF) [file pgen.1010910.s010.tiff]

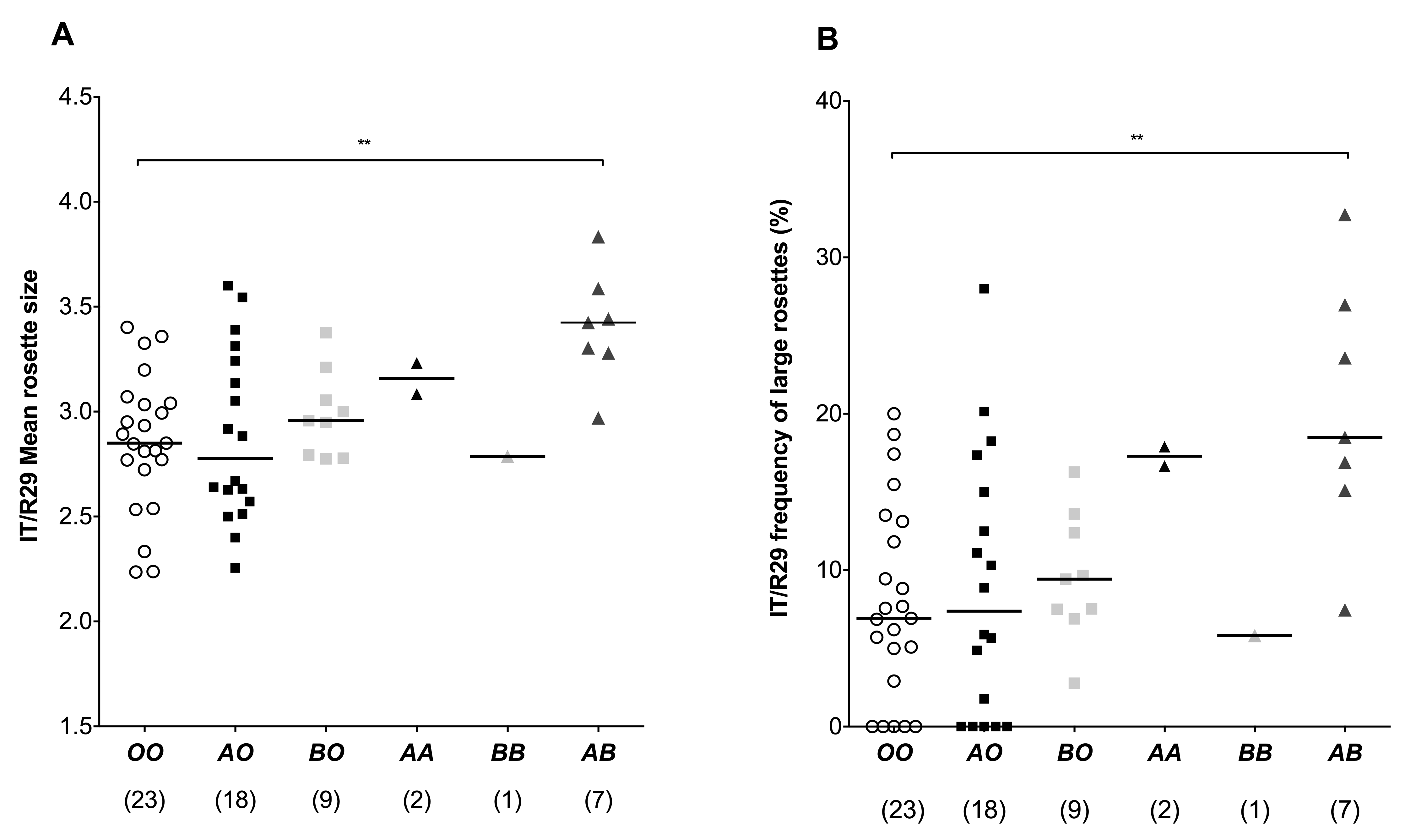

Supplement: S2 Fig — (A) IT/R29 mean rosette size (number of uninfected RBCs per rosette) (B) IT/R29 frequency of large rosettes (more than 4 uninfected RBCs per rosette). Purified IT/R29 infected RBCs (iRBCs) were allowed to invade into RBCs from 60 donors (OO n = 23, AO n = 18, BO n = 9, AA n = 2, BB n = 1, AB n = 7) and rosette size and frequency of large rosettes were assessed after one complete life-cycle by fluorescence microscopy. Samples were tested over two consecutive experimental days (day 1 = 30 and day 2 = 30) in duplicates. Horizontal bars represent the median rosette size and median frequency of large rosettes for each genotype. The number of donors per genotype are shown in parenthesis. Sample genotype was masked during counting to avoid observer bias. **AB differed from OO for both mean rosette size and frequency of large rosettes, p<0.01 Kruskal-Wallis test with Dunn’s multiple comparisons. (TIFF) [file pgen.1010910.s011.tiff]

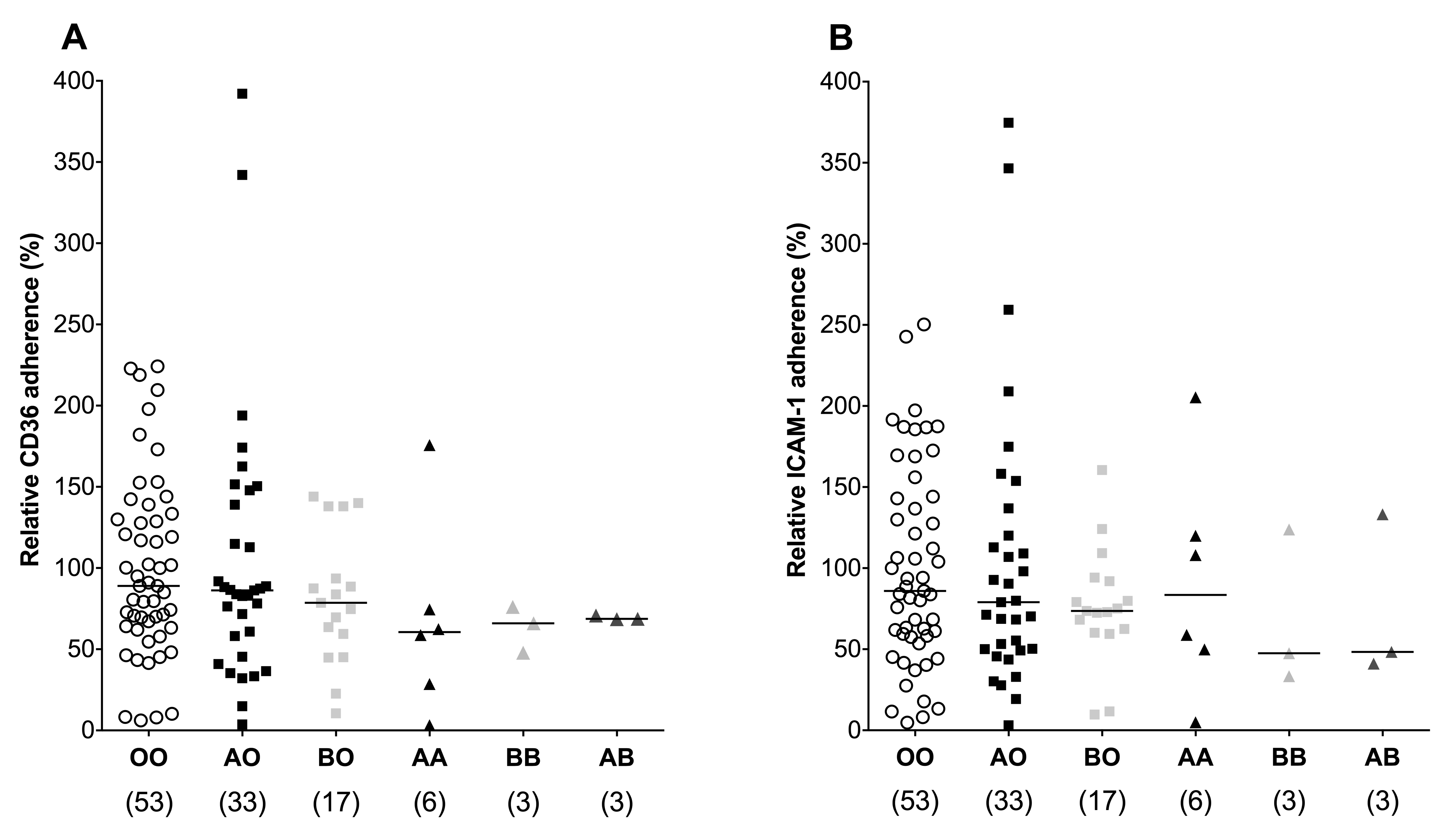

Supplement: S3 Fig — (A) Relative binding to CD36 recombinant protein. (B) Relative binding to ICAM-1 recombinant protein. Purified ITg infected RBCs (iRBCs) were allowed to invade into RBCs from 112 donors (OO n = 51, AO n = 32, BO n = 17, AA n = 6, BB n = 3, AB n = 3) and static adhesion to immobilized recombinant proteins was tested after one complete life-cycle. Samples were tested over seven experimental days (day 1 n = 13, day 2 n = 8, day 3 n = 12, day 4 n = 1, day 5 n = 55, day 6 n = 15 and day 7 n = 8), with each donor being tested once. For each RBC sample, adhesion was tested in two dishes with triplicate protein spots in each dish and the data presented as the mean iRBC bound/mm2 for each donor. Because baseline binding using a single donor varies from day to day, the binding data for each sample were normalized to that of the mean binding for the control iRBCs (OO) run on the same day (number of reference OO genotype samples run each day; day 1 n = 7, day 2 n = 4, day 3 n = 8, day 4 n = 1, day 5 n = 23, day 6 n = 7 and day 7 n = 2). Horizontal bars represent median relative adhesion for each genotype. Number of samples per genotype are shown in parenthesis. A Kruskal-Wallis test gave p = 0.310 for CD36 adhesion and p = 0.814 for ICAM-1 adhesion. (TIFF) [file pgen.1010910.s012.tiff]

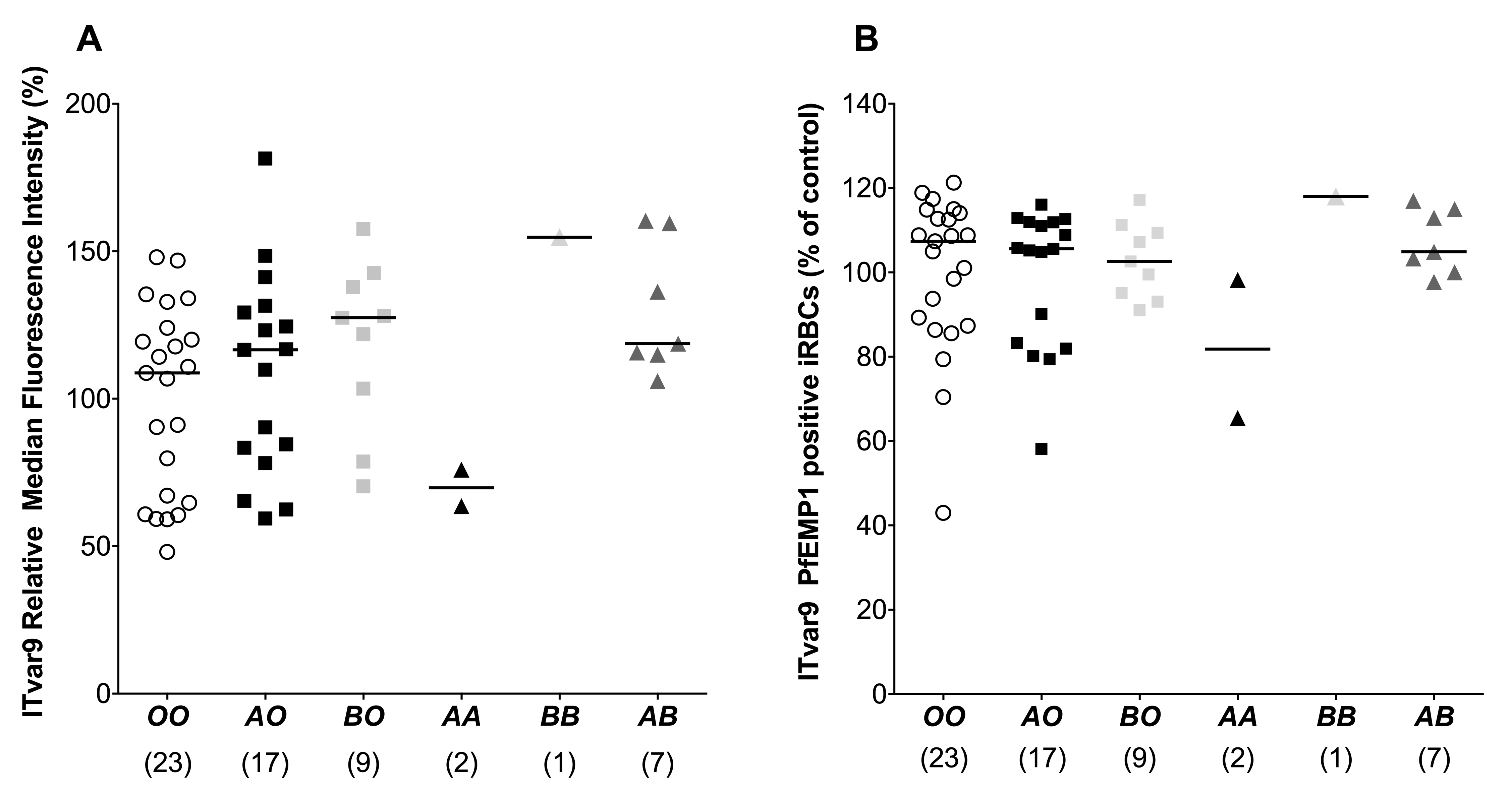

Supplement: S4 Fig — (A) Relative ITvar9 PfEMP1 expression. (B) Relative ITvar9 positive iRBCs. Purified IT/R29 infected RBCs (iRBCs) were allowed to invade into RBCs from 60 donors (OO n = 23, AO n = 18, BO n = 9, AA n = 2, BB n = 1, AB n = 7) and PfEMP1 expression was assessed after one complete life-cycle by flow cytometry with antibodies specific for the ITvar9 PfEMP1 variant. Samples were tested over two consecutive experimental days (day 1 = 30 and day 2 = 30). Median fluorescent intensity and proportion of ITvar9 positive iRBC data for all samples were normalized to that of the mean MFI and proportion of ITvar9 positive iRBCs for the control pRBCs (OO) (14 and 9 reference OO genotype samples run on day 1 and 2 respectively) run on the same day. Horizontal bars represent median relative MFI and proportion of ITvar9 positive iRBCs for each genotype. Kruskal-Wallis test p = 0.076 for MFI and p = 0.285 for % positive iRBCs. (TIFF) [file pgen.1010910.s013.tiff]
