# Supplementary material for: Non-O ABO blood group genotypes differ in their associations with Plasmodium falciparum rosetting and severe malaria
Source: PLoS Genet. 2023 Sep 14;19(9):e1010910. doi: 10.1371/journal.pgen.1010910 (PMC10522014; doi:10.1371/journal.pgen.1010910)
Supplement: Attachment — Submitted filename: 2_Opi et al_Response to reviewers_020623.pdf [file pgen.1010910.s014.pdf]

Responses to Reviewers' Questions

Comments to the Authors:
Please note here if the review is uploaded as an attachment.

We note that the review has been uploaded as an attachment.

Reviewer #1: Non-ABO blood group genotypes differ in their associations with Plasmodium falciparum rosetting and severe malaria
This work described tackles a key question in malaria pathogenesis, does ABO blood type influence clinical features of disease? The work is focused partially around determining an association between Non-O dosage and clinical outcome/risk of severe disease and partially around investigating an association between ABO blood type and rosetting, a feature of malaria infection where parasitized RBCs will "coat" themselves in uninfected RBCs. Overall, this work is interesting and exciting to the field. The results are conveyed with clarity, and the caveats regarding the work well highlighted. Of note is the association study which ultimately stops short of demonstrating an association between O-dosage and the outcome of infection, though replicates a link between O vs non-O, and the link between rosette size and non-O dosage.
The major unique factor of the work, to my eye, is the focus on non-O dosage. The advance described is arguably subtle, showing a tendancy for non-O dosage to increase adverse outcome in the association study. As noted, a lack of sample size and numbers in some ABO genotype groups reduced the power of the study.
As I understand the work, there is a link between O dosage and either rosetting or clinical outcome, beyond which has already been reported (i.e. O vs the rest). This is the result of hard won and clearly described data and analysis and is supportive of a role whereby (1) non-O genotype dosage influences the clinical outcome of malaria disease and (2) rosetting phenotypes vary between ABO genotypes. While this appears to be a subtle advance, the careful work described here is robust and should be a springboard for larger studies with better power.

Given the small numbers in Figure 1, and the focus of the paper around non-O dosage it was somewhat surprising to not see the grouping by number of non-O alleles.

We have replaced Figure 1 with a graph showing the data grouped by the number of non-O alleles, as suggested by the reviewer, and we have modified the description of the data in results lines 416-426. We have moved the previous Figure 1 showing each genotype separately to the supplementary information (Figure S2). These changes make no material difference to the conclusions of this study, but we hope they provide greater clarity for readers.

See also the response to reviewer 3, point 18 below for further comment on the rosetting data.

The derivation of the IT clones for growth in A+ cells could have been given greater discussion. The IT/R29 clone, it appears that two clones are used in the study (R29 expressing ITvar9 PfEMP1 and another (ItG), clone expressing ITvar16). How may the way these lines were derived impact the findings of the study? These lines, as I understand, were derived through selection under specific conditions. How stable are the phenotypes in each of these clones, and was the phenotype of each line confirmed using controls?

We can confirm that both the IT/R29 and the ItG parasite clones were originally derived from the parental strain IT4/25/4 as described in Roberts et al, Nature 1992, PMID: 1614515. Due to antigenic switching, both strains do gradually lose their respective adhesion phenotypes in long-term culture. For our study, we repeatedly selected IT/R29 for rosetting using standard methods of gelatin flotation and density centrifugation to maintain the line at >50% rosette frequency (Handunnetti et al, AJTMH 1992, PMID: 1575284), while ItG was selected for ICAM-1 binding by repeated panning on ICAM-1 coated dishes and then frozen in aliquots. For the experiments described here, an aliquot of ItG was thawed for culture and used for cytoadhesion experiments within 3 weeks. We have added these details to the methods lines 255-261.

A minor aside; Kappa score is described as 100% to 0% in the methods, and 0.95 listed in the results. I assume that either the score should be described as a proportion of 0 to 1 or the results should be 95%

We thank the reviewer for pointing out this error. We have corrected the methods line 198 to reflect the Kappa score range of 0 (no agreement) to 1 (complete agreement).

Reviewer #2: Manuscript Number: PGENETICS-D-22-01112
Full Title: Non-O ABO blood group genotypes differ in their associations with Plasmodium falciparum rosetting and severe malaria

Summary: This observational study of Kenyan children from a hospital-based case-control study and a community-based cohort examines the associations between genetically-determined ABO genotypes and risk of severe malaria. These associations are supported by in vitro assays of rosette formation in RBCs of different ABO types infected with laboratory strains of P falciparum. While this study does not necessarily present new biology about malaria pathogenesis, it does improve the precision of our understanding of the relationship between ABO genotypes and rosetting phenomena. While the experimental and epidemiological methods are sound, more attention to the interpretation of multiple testing needs to be provided.

Major comments:
1. Rosettes are proposed to contribute to severe malaria pathogenesis by obstructing microvessels. Presumably, rosettes would be susceptible to removal from the circulation by the spleen. Were there any associations between rosetting genotypes and measures of anemia?

We are unable to address this question with our dataset because we do not have rosette frequency data for the parasites infecting the children in the case-control study. There are, however, some insights from the literature that are relevant. Firstly, rosetting parasites are thought to contribute to sequestration and do not circulate freely in the peripheral blood (as evidenced by experiments with ex vivo microvasculature eg. Kaul et al, Blood 1991 PMID: 1859893; imaging of human tissues eg. Scholander et al, Nat Med 1996 PMID: 8574966 and *in vitro* experiments showing PfEMP1 variants with dual rosetting and cytoadhesion phenotypes eg. Adams et al, Infect Immun 2014 PMID: 24343658). Secondly, previous studies have shown significant associations between parasite rosette frequency and malarial anaemia (eg. Newbold et al, AJTMH 1995 PMID: 9347951; Rowe et al, AJTMH 2002 PMID: 12224576; Doumbo et al, AJTMH 2009 PMID: 19996426) and a possible mechanism for this has been described involving oxidative damage to the uninfected erythrocytes in rosettes (Uyoga et al, Br J Haematol 2012 PMID: 22352722). Given the length of the manuscript, we have not added additional discussion points around this topic, but we would be happy to do so if the reviewer thinks this would improve the work.

2. It is disconcerting to observe non-random associations between ABO genotypes encoded on chromosome 9 and HBA and HBB genotypes encoded on chromosomes 16 and 11 in the population cohort described in Table S5. Are these spurious associations? If not, what is the reason for these associations? Why the age differences by ABO genotypes?

We think it likely that these associations are spurious due to the rarity of the homozygous ABO genotypes (AA n=2, BB n=4, AB n=5) in the longitudinal cohort (n=242). We have added a footnote to this effect to the Table (which is Table S7 in the revised manuscript).

3. In the case-control study described in Table 3, why is ABO genotype associated with sex and age? Perhaps these associations are driven by cases due to relationships with susceptibility to malaria? If so, perhaps the cases and controls need to be analyzed separately, either way an explanation for these unexpected associations needs to be offered.

To answer these queries, we have replaced Table 3 with two tables (Table 3 and Table S1). Table 3 now gives the general demographic characteristics of the cases and controls, analysed separately. Table S1 gives the distribution of ABO genotypes in relation to assorted variables including gender and age, in cases and controls analysed separately.

Table 3 shows that cases and controls differ significantly in ethnic composition, hence the inclusion of ethnicity as a variable in the logistic regression analysis. This table also highlights the age differences between cases and controls, which is a consequence of our study design. The issue of age was also raised by reviewer 3 (major comments #2 and #4) and we apologise for the lack of clarity on this issue in our original manuscript. To answer these age-related queries, we have added a paragraph to the methods lines 146-152 (shown below) to explain the rationale behind the study design.

"Controls (n=3949) were healthy children who were born within the study area between August 2006 and September 2010 and who were recruited at 3-12 months of age into a genetics cohort study (48). Therefore, controls were matched to cases by location but differed from cases in age-structure. While not typical of classical case-control design, cord blood or infant samples have been widely used as controls in previous genetic association studies conducted across sub-Saharan Africa (12, 13, 49), because this provides the most feasible way of collecting large sample numbers in resource-limited settings."

A potential disadvantage of this approach is that differential mortality by ABO genotype among children could potentially give rise to a changing prevalence by age. However, we do not believe that this represents a significant problem in the interpretation of this and similar studies because (1) the overall rates of childhood mortality during the period were too low (<1%) to make a material difference and (2) any such effect would lead to a rising frequency of protective genotypes with age and, as such, the use of young children as controls might reduce the power of the study to discover malaria-protective associations but would not give a bias towards false positive results.

In the revised Table S1 it can be seen that when the data are shown for cases and controls separately, there are no significant differences in the prevalence of the different ABO genotypes by age group among the cases. We are unsure why this result differs from that shown in our original submission, which combined cases and controls, but assume this was a chance finding. For gender, there is no significant difference in the cases, but in controls, females differ from the males in ABO genotypes distributions (p=0.025). The biological

significance of this is unclear and would require replication. For the purposes of this manuscript, gender was adjusted for in the logistic regression analysis.

4. Some acknowledgement, discussion, or statistical adjustment should be made regarding multiple tests performed and the interpretation of p values.

For the *in vitro* experiments, we used Dunn's multiple comparisons test which adjusts for multiple comparisons. This information has been added to the methods lines 317-318.

For the epidemiological data, additional text has been inserted into the methods to explain our approach, lines 233-238, shown below:

"In the epidemiological analyses, adjustments for multiple testing were not performed; instead all adjusted odds ratios, confidence intervals and *p* values have been clearly reported and it is emphasized that no single study is conclusive and additional studies are needed to determine if results are replicable. This approach has been suggested previously for epidemiological data, to avoid potentially important findings being discarded (type II error) due to the stringency of multiple comparison adjustments (eg. Rothman, Epidemiology 1990 PMID: 2081237; Perneger, BMJ 1998 PMID: 9553006; Nakagawa, Behavioural Ecology 2004; Rothman, J Gen Intern Med 2014 PMID: 24452418)."

Minor comments:
5. While the statement in line 352 may be true, "To the best of our knowledge, whether host RBC ABO genotype influences P. falciparum rosetting has not been investigated previously." It is really a stretch especially when you show such a high concordance between ABO genotype and phenotype.

We have removed the above statement and adjusted the wording to read "To determine whether host RBC ABO genotype influences *P. falciparum* rosetting, we examined rosette size following parasite invasion into RBCs from 60 donors…." lines 414-415.

Typos, etc:
6. The subheading in line 298 could be stated in the past tense.

This has been updated accordingly to reflect the past tense (line 348).

Reviewer #3: This study aimed to analyze the association between ABO blood group genotype and severe malaria in Sub-Saharan Africa. Expanding on previous knowledge that individuals with blood type O are less vulnerable to severe malaria than non-O blood types, the authors hypothesized that "double-dose" genotypes (AA, BB, or AB) would be more vulnerable to severe malaria compared to "single-dose" genotypes (AO, BO, or OO). This is an interesting and important hypothesis that could possibly improve the tailoring of severe malaria treatment and prevention. The manuscript is well-written and straight-forward to follow. The most valuable contribution is the data analysis from a large case-control study in Kenya, finding that children with double-dose genotypes versus single-dose genotypes had higher odds of severe malaria over children with the OO genotype. Importantly, this was also true for separate severe malaria syndromes. However, it will be crucial to see if these results remain robust after age-adjustment. It was also important to learn that ABO genotype may not always concur with phenotype, which should be of interest to the broader field of

genetics and genomics.

The authors included additional epidemiological and mechanistic analyses with a more limited contribution to the overall findings. These analyses had small sample sizes and possible statistical overfitting, raising concerns that this in vitro evidence was not substantial enough to support the article's conclusions.

Major comments

1. The authors' hypothesis is that double-dose genotypes (AA, BB) are more vulnerable to severe malaria than single-dose genotypes (AO, BO). The authors use Wald tests to determine that the double-dose genotypes had higher odds of severe malaria over OO compared to the single-dose genotypes, but they do not find statistical significance. Why not compare the odds of severe malaria between double-dose and single-dose genotypes directly?

We thank the reviewer for this suggestion, and we did initially consider analysing the data in the manner they suggest. However, after consultation with statistician colleagues, we concluded that there were benefits to using logistic regression to generate odds ratios compared to the *OO* genotype (allowing easy comparison to all prior studies on ABO blood groups and malaria) and then using the Wald test to examine differences between single- and double-dose non-*O* genotypes (with the advantage of consistency, comparing odds ratios generated by comparison to the same reference group across all non-*O* genotypes).

However, to fully answer this query, we have now carried out the analysis suggested by the reviewer, the results of which are shown below. It can be seen from the table that double dose non-*O* genotypes are associated with increased odds of severe malaria compared to single dose non-*O* genotypes in all cases (i.e. odds ratios of >1), but that these associations were only statistically significant when looking at the comparison between *AB* and *AO* genotypes. This mirrors the results shown in Table 5 for the Wald test. We have added this table to the supplementary information (Table S4) and described the additional analysis in the methods (lines 215-218) and provided brief comment in the relevant results section (lines 404-409).

Table S4. A comparison of the odds ratio differences for severe malaria between single dose and double dose *non-O* genotypes using logistic regression with single dose *non-O* genotypes *AO/BO* as reference.

			Crude				Adjusted†			
**Case Phenotype**	**No. Cases/controls**	***ABO* genotype**	**OR**	**LCI**	**UCI**	***p* value**	**OR**	**LCI**	**UCI**	***p* value**
All SM	306/810	*AO*	1				1			Reference
	37/83	*AA*	1.18	0.78	1.78	0.428	1.15	0.74	1.78	0.528
	61/115	*AB*	1.40	1.00	1.97	0.049	1.53	1.07	2.18	0.020
All SM	337/683	*BO*	1				1			Reference
	34/55	*BB*	1.25	0.80	1.96	0.323	1.26	0.77	2.07	0.351
	61/115	*AB*	1.08	0.77	1.51	0.674	1.17	0.82	1.68	0.384

Double dose *non-O* genotype odds ratios for severe malaria were generated following a fixed-effects logistic regression model comparing genotype frequencies between the non-*O* double dose genotypes (*AA/AB* or *BB/AB*) to the reference *non-O* single dose genotypes (*AO* or *BO*) with adjustments for self-reported ethnicity, gender, α^{+}thalassaemia and HbAS. SM: Severe malaria; OR: Odds Ratio; LCI: Lower Confidence Interval (95%); UCI: Upper Confidence Interval. †Adjusted for HbS, α+thalassaemia, gender, ethnicity and interaction (HbS and α+thalassaemia).

2. In the case-control study, are there age differences between the cases and controls? I would like to see the demographic characteristics stratified by cases/controls. It seems that age is not controlled for and, given that there are age differences between the genotypes, it may be an important confounding factor to consider. Ref 14 suggests that there may be a significant age difference between cases and controls. The authors did control for age in the cohort and cross-sectional analyses.

As discussed in our response to a similar point made by reviewer 2, for methodological reasons the controls were significantly younger than the cases. We have modified Table 3 to show the demographic characteristics stratified by cases and controls as requested, and we have added text to the methods to explain the use of controls that differ in age from cases. Please see the response to reviewer 2 (comment 3) above for full details.

3. Were there any episodes of severe malaria in the controls? This information should be stated.

We have added the information that all controls were healthy children at the time of recruitment (methods line 146).

4. In Table 4, the authors used the same controls for each severe malaria phenotype comparison. Again, this makes me wonder whether the control pools are age-comparable to different subsets of cases.

Please see the response to reviewer #2 comment 3 above that explain the use of controls that differ in age from cases.

5. In Table 5, the accompanying results text, and the discussion, the authors should be clear that the reference group is still OO in all odds ratios presented. Rather than comparing single-dose to double-dose genotypes directly, they are comparing the magnitude of each genotype to OO.

To emphasize that the odds ratios used in the Wald test were generated by comparing non-*O* genotype frequencies to the *OO* reference genotype frequency in the logistic regression analysis we have added a footnote to Table 5 and additional text in the results lines 394-401 and the methods lines 210-215.

In response to major comment 1 above we have also added a supplementary table (Table S4) showing the odds ratios from logistic regression analysis comparing double dose to single dose non-*O* genotypes.

6. The sample sizes of RBCs for the in vitro rosetting and receptor adhesion assays were very small for the AA, BB, and AB genotypes, reducing the reliability of the statistical findings. The authors should qualify their conclusions about static adhesion and PfEMP1 expression, as their findings are limited to two PfEMP1 variants (particularly Lines 415-416).

We have added text to the discussion on rosetting, cytoadherence and PfEMP1 expression acknowledging this limitation in our study and proposing future studies with increased sample sizes for the key genotypes (lines 473-477 and 485-491).

We have also updated Figure 1 to show the rosetting data for single dose and double dose genotypes in response to reviewer 1's suggestion, and modified the description of these data in the results lines 416-426.

7. Consider changing Line 423 to "a key mechanism", not "the key mechanism"

This change has been made (line 498).

8. Related to above, the authors adjusted the in vitro signals for several covariates, but I am not sure that the sample size can support so many parameters without overfitting the model.

In the initial analysis we explored a number of potential confounding variables that have published associations with the *in vitro* parasite adhesion phenotypes being studied here including HbS and α^+thalassaemia genotypes, mean corpuscular volume, complement receptor 1 level and Knops blood group. Each variable was tested in a univariate analysis and only variables that showed significant associations ($p<0.05$) were included in the final multivariate model. Additionally, variables were examined for their overall improvement of model fitness using the loglikelihood ratio (LR) test, and only variables that significantly improved the model fitness were included in the final model (LR test p value <0.05). Using this criterion only HbS and/or α^+thalassaemia were included and adjusted for in the final models. Therefore, it is unlikely that that regression outputs reported here represent noise due to overfitting the regression model, but rather represent true relationships between *ABO* genotypes and the specific predictor variables.

To clarify this approach, the methods lines 307-314 have been edited to more fully describe the testing and inclusion of confounding variables (HbAS and/or α^+thalassaemia) in the final model. Additionally, the exact details of confounding variables included in each analysis have been added to the footnote for each results table.

Finally, we note that the outputs from the multivariate analyses of rosetting data were consistent with the simple non-parametric analyses shown in the graphs in Figure 1 and Figure S2. Overall, the rosetting assays tested red blood cells from 60 donors in blinded experiments and we are confident that the analyses and conclusions based on these data are sound. However, as described in response to comment 6 above, we have emphasized that small numbers in the key "double dose" genotypes is a limitation of this study, and as always with biological data, further studies will be needed to replicate the findings.

9. While the ABO findings in the cohort study are interesting, they would be more informative if there was specific information about rosetting in the symptomatic and asymptomatic episodes. As such, the conclusions from this section appear limited and somewhat peripheral to the rest of the study.

Unfortunately, no parasite material was collected in the cohort study to address this question. We agree that the cohort study is of lesser interest than the case-control study, and this is reflected by the inclusion of the cohort study data in the supplementary information rather than the main body of the manuscript. However, the cohort study does examine a different aspect of the relationship between *ABO* genotypes and malaria i.e. uncomplicated and asymptomatic infection, and as such, we think the data will be of interest to some readers and merit inclusion in the manuscript.

10. Are there instances of clinically significant ABO genotype-phenotype discrepancies? As this is a motivating concern underlying this study, any examples should be cited and explained in the Introduction and/or Discussion.

We are not aware of clinically significant *ABO* genotype-phenotype discrepancies, but we were unwilling to accept the assumption that the *ABO* genotype-phenotype relationships described in Caucasian populations necessarily apply in sub-Saharan Africa, in the absence of any data addressing the topic. Our study now provides these data, and thus validates the prior studies of ABO and malaria that are based on this assumption.

We can provide an example of a different gene (*CR1,* encoding complement receptor 1) where genotype-phenotype relationships in Caucasians were mistakenly assumed to apply in sub-Saharan Africa, leading to erroneous conclusions. The *CR1* gene has SNPs that correlate with erythrocyte CR1 expression level in Caucasians. A genetic association study examined whether these SNPs were associated with severe malaria in West Africa (Bellamy *et al*, 1998 Tran Roy Soc Trop Med Hyg PMID: 9861406) and concluded that low erythrocyte CR1 level was not associated with severe malaria. However, later work showed that the *CR1* SNPs, although present in African populations, do not correlate with erythrocyte CR1 expression levels in Africans (Rowe *et al*, Gene Immun 2002, PMID: 12486610). Hence, the discrepancy in *CR1* genotype vs CR1 phenotype meant that the conclusions of Bellamy *et al* were flawed. Given the length and complexity of our manuscript, we have not added this example to the discussion, but we would be happy to do so if the reviewer thinks it is a useful addition.

11. The Discussion should comment on the specific findings for particular severe malaria syndromes and ABO genotypes.

We have included a minor comment on the specific severe malaria syndromes in the Discussion paragraph that emphasizes the limitations of the study in relation to sample size (line 467-477). Given that these acknowledged limitations are particularly applicable to the specific severe malaria syndromes, we were reluctant to speculate on these results in further detail at this point.

Minor comments

- Background

12. It should be mentioned that RIFINs and STEVORs also mediate rosetting, especially RIFINs in blood type A.

We have added text lines 82-85 describing the role of RIFINs and STEVORS in *P. falciparum* rosette formation and included relevant references.

13. The cohort and cross-sectional analyses were not introduced.

Text introducing the cohort and cross-sectional studies has been added (lines 118-120).

- Results

14. I don't understand what the authors were trying to show by demonstrating Hardy-Weinberg Equilibrium among the controls in the case-control study. This should be clarified.

Assessment of Hardy Weinberg Equilibrium (HWE) among control samples is a quality control step in genetic association studies (eg. MalariaGen et al, Nature Genetics 2014 PMID: 25261933; Namipashaki et al Cell J 2015 PMID: 26199897). Departure from HWE in control samples reflects underlying problems with the data such as genotyping errors or selection bias, such that any SNPs displaying large deviations from HWE ($p<0.05$) in controls are routinely excluded from further analysis. The ABO genotype SNPs studied in this manuscript did not show departure from HWE in controls.

We have added text and references in the methods lines 188-194 to clarify this point, and also provided the relevant HWE data for the study in this same paragraph.

15. Reading the text, I was wondering what an example of ABO genotype-phenotype discordancy may look like. It was made clearer in Table 2 and the discussion, but an example in the text would help.

We have added text to the results section (lines 360-362) to rectify this omission, giving the example of *BO* and *BB* genotypes and blood group B phenotype that show the highest level of discordancy (~7%). The possible reasons for this discordancy are described in detail in the discussion section (lines 508-521).

16. In Table 3, I think that column percentages would support the authors' argument better than row percentages.

We have now updated Table 3 and added a new supplementary table (Table S1) in response to all the reviewers' comments, and we hope that this revised form of presentation provides greater clarity for readers.

17. The authors should consider providing interpretation of the odds ratios from Table4. For example, they could indicate that non-O genotypes had 49% higher odds of severe malaria compared to OO. Simply stating that "the odds ratio is 1.49" does not help the reader's understanding.

We have updated the results lines 386-401 to include interpretation of the odds ratios as suggested by the reviewer.

18. Since the authors used a parasite strain that preferentially forms rosettes with blood type A, for their rosetting experiments, it seems like the only important result is that AA > AO. Could they derive information about B-antigens if they did not use an A-preferring parasite?

The IT/R29 line is typical of many blood group A-preferring parasites (similar examples are given in Udonsangpetch et al, AJTMH 1993 PMID: 8447516) in that it shows a strong blood group A preference, but also shows a lesser tendency towards preferring group B over group O (in our pilot experiment shown in Figure S1, this trend did not reach statistical significance when analysed by conservative non-parametric methods). Another published example of this phenomenon is the VarO parasite, in which the blood group preference is A>B>O, and in which the parasite adhesion molecule PfEMP1 binds both A and B antigens but has a higher affinity for A (Vigan-Womas et al, PLoS Pathog 2012 PMID: 22807674).

Therefore, we think there is value in analysing the rosetting data in relation to all *ABO* genotypes, and we apologise for the lack of clarity in describing this in the original

manuscript. In the revised manuscript we have modified the results lines 416-426 to describe the blood group preference of IT/R29 more fully and provided the data for all genotypes in Table 6 and Figure S2, while also providing grouped single dose and double dose genotype data in Figure 1, in response to the other reviewers comments.

We hope that providing the data for individual genotypes and for single vs double dose genotypes in both the epidemiological and rosetting parts of the study gives readers the information required for them to judge the data fully.

We did originally intend to carry out parallel rosetting experiments with a parasite line showing a strong blood group B-preference. However, the TM284 rosetting strain that is published as a B-preferring line (Carlson et al, Blood 1992 PMID: 1402677) did not show a preference for group B in our hands in a pilot experiment. Hence, we were unable to carry out the rosetting experiments for a strong blood group B-preferring line.

19. The authors should expand on the null findings for the cytoadhesion and cohort study sections in the Discussion, with specific acknowledgment of limitations and next steps.

As described in the response to point 6 above, we have provided further text in the Discussion lines 473-477 and 485-491 to emphasize that small sample size in key double dose genotypes is a limitation for both the epidemiological studies and the in vitro assays, and that further work will be needed to replicate the findings described here.

Have all data underlying the figures and results presented in the manuscript been provided?
Large-scale datasets should be made available via a public repository as described in the *PLOS Genetics* data availability policy, and numerical data that underlies graphs or summary statistics should be provided in spreadsheet form as supporting information.

Reviewer #1: Yes

Reviewer #2: Yes

Reviewer #3: **No:** The datasets from the case-control study and cohort study are not included.

These datasets are available at https://doi.org/10.7910/DVN/A5KILM and can be accessed on request from the KEMRI-Wellcome Trust Research Programme Data Governance Committee (dgc@kemri-wellcome.org) (lines 555-558).

PLOS authors have the option to publish the peer review history of their article (what does this mean?). If published, this will include your full peer review and any attached files.

If you choose “no”, your identity will remain anonymous but your review may still be made public.

Do you want your identity to be public for this peer review? For information about this choice, including consent withdrawal, please see our Privacy Policy.

Reviewer #1: No

Reviewer #2: No

Reviewer #3: No
